# Supplementary material for: Overcoming Poor Transgene Expression in the Wild-Type Chlamydomonas Chloroplast: Creation of Highly Mosquitocidal Strains of Chlamydomonas reinhardtii
Source: Microorganisms. 2022 May 25;10(6):1087. doi: 10.3390/microorganisms10061087 (PMC9229432; doi:10.3390/microorganisms10061087)
Supplement: Supplementary file 1 [file microorganisms-10-01087-s001.zip › microorganisms-1721305-supplementary.pdf]

Table S1. Sequences of primers used in this work

| ID no. | Name | Sequence |
|---|---|---|
| 799 | Cry11A F | GGAAGACTCATCATTAGACAC |
| 800 | Cry11A R | AGTAGCAGTGTTGAAACCAGT |
| 847 | psbD5’ F | gctcccatggatccTCATAATAATAAAACCTTTATTCAT *NcoI BamHI* |
| 850* | psbD5’ R | ccggcatatgGTGTATCT**TT**AAAATAAAAAAACAACTCATCGTTACG *NdeI* |
| 860 | psbA3’ F | cggggctgAGCTCAAACAACTAATTTTTTTTTAAAC *BlpI* |
| 861 | psbA3’ R | cagtgctcagcggaTCCTGCCAACTGCCTATGGTAGC *BlpI BamHI* |
| 863 | p322 ins ups F | TAACCCATAAATAGTTTCAATTGGAA |
| 880 | rbcL5’ F | aataccatggatccATATACCTAAAGGCCCTTTCTAT *NcoI BamHI* |
| 881 | rbcL5’ R | ccggcatatgTATAAATAAATGTAACTTCTTTTGACG *NdeI* |
| 882 | rbcL3’ F | tcaggctgagcTTGCTCGTGAAGGTGGCGACG *BlpI* |
| 883 | rbcL3’ R | taatgctcagcggatccCACAACCTCGAACTTGGAAGGA *BlpI BamHI* |
| 975 | p322 ins dns R | AATGCAAAGTACCATCAGATATTGCTA |
| 997 | rps4 P+5’ F | gctcccatggatccTCGTCCACTCTAATGAGTTAATATA *NcoI BamHI* |
| 998 | rps4 P+5’ R | ccggcatatgAATTTTTTATTATTTTAAATAAACGGCTT *NdeI* |
| 1002 | Cry11A RT F | AGACTTCTACTCAAAAAAATCACACTAC |
| 1004 | Cry11A RT R | GAGAAAGCAGCTTTAGCAGCCCATTT |
| 1007 | 16S P F #2 | gctcccatggatccTTATTTTACTGCGGAGCAGCTTG *NcoI BamHI* |
| 1008 | p655 ins dns R | CCAAATTATATTTGTCGTCCACGAG |
| 1009 | p655 ins ups F | CCTCCTAACGGAGCATTAAAATC |

1020 16SmP rps4 5’fusion R
ccatatcgagcatatgAATTTTTTATTTATTTTAAATAAACGGCTTTAATGAATTAAC*TTTTTAATTTAACTTTTTTATTTTTTTTTGTC* *NdeI* italics indicate 16SmP portion

1021 psbD P rps4 5’fusion R
ccatatcgagcatatgAATTTTTTATTTATTTTAAATAAACGGCTTTAATGAATTAAC*ATTTAATATTATAACTTATATAAAATAAAATTAAAAATAAGC* *NdeI* italics indicate psbD P portion

1022 16SnP rps4 5’fusion R
ccatatcgagcatatgAATTTTTTATTTATTTTAAATAAACGGCTTTAATGAATTAAC***G****TTTTTAATTTAACTTTTTTATTTTTTTTTGTC* *NdeI* italics indicate 16S P portion; bolded G is the last nt of the 16S P (compl.), missing in #1020

* For primer #850, the bolded T’s indicate a change from C to destroy the Shine Dalgarno-like sequence (complement is shown).
